# Seroprevalence of Antibodies against *Plasmodium falciparum* Sporozoite Antigens as Predictive Disease Transmission Markers in an Area of Ghana with Seasonal Malaria Transmission

**DOI:** 10.1371/journal.pone.0167175

**Published:** 2016-11-22

**Authors:** Kwadwo A. Kusi, Samuel Bosomprah, Eric Kyei-Baafour, Emmanuel K. Dickson, Bernard Tornyigah, Evelina Angov, Sheetij Dutta, Daniel Dodoo, Martha Sedegah, Kwadwo A. Koram

**Affiliations:** 1 Department of Immunology, Noguchi Memorial Institute for Medical Research, College of Health Sciences, University of Ghana, Legon, Accra, Ghana; 2 Department of Biostatistics, School of Public Health, College of Health Sciences, University of Ghana, Legon, Accra, Ghana; 3 Malaria Vaccine Branch, Walter Reed Army Institute of Research, Silver Spring, MD, United States of America; 4 Malaria Department, Naval Medical Research Center, Silver Spring, MD, United States of America; 5 Department of Epidemiology, Noguchi Memorial Institute for Medical Research, College of Health Sciences, University of Ghana, Legon, Accra, Ghana; Université Pierre et Marie Curie, FRANCE

## Abstract

**Introduction:**

As an increasing number of malaria-endemic countries approach the disease elimination phase, sustenance of control efforts and effective monitoring are necessary to ensure success. Mathematical models that estimate anti-parasite antibody seroconversion rates are gaining relevance as more sensitive transmission intensity estimation tools. Models however estimate yearly seroconversion and seroreversion rates and usually predict long term changes in transmission, occurring years before the time of sampling. Another challenge is the identification of appropriate antigen targets since specific antibody levels must directly reflect changes in transmission patterns. We therefore investigated the potential of antibodies to sporozoite and blood stage antigens for detecting short term differences in malaria transmission in two communities in Northern Ghana with marked, seasonal transmission.

**Methods:**

Cross-sectional surveys were conducted during the rainy and dry seasons in two communities, one in close proximity to an irrigation dam and the other at least 20 Km away from the dam. Antibodies against the sporozoite-specific antigens circumsporozoite protein (CSP) and Cell traversal for ookinetes and sporozoites (CelTOS) and the classical blood stage antigen apical membrane antigen 1 (AMA1) were measured by indirect ELISA. Antibody levels and seroprevalence were compared between surveys and between study communities. Antibody seroprevalence data were fitted to a modified reversible catalytic model to estimate the seroconversion and seroreversion rates.

**Results:**

Changes in sporozoite-specific antibody levels and seroprevalence directly reflected differences in parasite prevalence between the rainy and dry seasons and hence the extent of malaria transmission. Seroconversion rate estimates from modelled seroprevalence data did not however support the above observation.

**Conclusions:**

The data confirms the potential utility of sporozoite-specific antigens as useful markers for monitoring short term/seasonal changes in malaria transmission. It may however be essential to update models to allow for assessment of seasonal changes in malaria transmission, which usually occur within four to six months.

## Introduction

Monitoring of malaria transmission has become very crucial as a number of endemic countries continue to make tangible gains in disease control. According to the WHO, an increasing number of these countries are approaching the disease elimination phase [1]. Current standard tools for monitoring malaria transmission are largely insensitive and laborious to undertake in areas with low transmission and this presents a unique challenge to control and elimination programmes since a lapse in monitoring can easily result in a resurgence of malaria. The development of more sensitive monitoring tools will therefore be an essential component of control strategies.

*Plasmodium falciparum* infection elicits antibody responses to multiple stages of the parasite and the presence of these antibodies in the blood of persons with current and previous infections can be used as a marker of parasite exposure. Antibodies to parasite antigens such as apical membrane antigen 1 (AMA1), the 19 kDa fragment of merozoite surface protein 1 (MSP1_19_) and merozoite surface protein 2 (MSP2) have all been used as biomarkers of malaria transmission in various endemic settings [2–4]. In areas of stable medium to high transmission, estimates of anti-AMA1, anti-MSP1_19_ and anti-MSP2 antibody seroconversion rates from modeled cross-sectional data correlate well with standard entomological inoculation rate (EIR) estimates [2,3]. A recent sero-epidemiological study that utilized two longitudinal cohorts, the first with a one year follow up and the second with four years of follow up, identified three parasite antigens that could predict an individual’s exposure to parasites within 30, 90 or 365 days [5]. In addition, this study also identified six other parasite antigens, including CSP and MSP2, which effectively predicted the incidence of malaria in the preceding year [5]. These sero-epidemiological approaches to transmission estimation have an advantage over parasite prevalence estimation approaches since antibody decay is slower than parasite clearance rates. A downside to sero-epidemiological transmission estimation is however the persistence of antibodies long after transmission has ceased [6–8].

The sporozoite stages of *P*. *falciparum* are exposed to the immune system for a relatively short time after an infectious bite, compared to the asexual blood stage infection which can persist for much longer times, especially in asymptomatically infected individuals. Sporozoite-specific antibody levels have been shown, at least in an experimental human challenge study with sporozoite immunization, to increase with each immunization/challenge and declined in the absence of parasite exposure between immunization and challenge [9]. The levels of antibodies against sporozoite stage antigens are therefore expected to be relatively higher in individuals with frequent or recent exposure to infectious bites and lower in those with no recent exposure. We have previously shown in an area of very low malaria transmission that antibodies to two such sporozoite antigens, circumsporozoite protein (CSP) and cell traversal for ookinetes and sporozoites (CelTOS), indeed had relatively higher decay rates compared to the classical blood stage antigen AMA1 [10]. Reversible catalytic models based on seroprevalence of antibodies to CSP further predicted a 13-fold decrease in seroconversion rate and hence malaria transmission, an event which occurred approximately four years prior to the time of sampling in this area [10]. The association between anti-CSP antibodies and malaria transmission intensity as estimated by such modeled cross-sectional data are consistent with similar estimates based on longitudinal data [11–14].

It is however unclear how antibody seroconversion estimation models, which estimate yearly seroconversion rates based on cross-sectional antibody seroprevalence data, will be able to effectively predict short term changes in transmission that are observed within four to six months in areas of seasonal malaria transmission [15,16].

In the current study, we sought to evaluate a disease transmission intensity estimation model based on anti-sporozoite antibody seroprevalence for predicting short term changes in malaria transmission intensity in two communities in the Bongo District of the Upper East region of Ghana where malaria transmission is highly seasonal. Both communities have had a high penetration of malaria intervention tools such as insecticide-treated mosquito nets and intermittent treatment of malaria in pregnancy, either through programmes of the national health system or through studies that have been undertaken in the district for over two decades [17–19]. One of the study communities, Gowrie/Vea, is in close proximity to the Vea irrigation dam while Soe, the other study community, is at least 20 Km away from the Vea dam. We therefore also sought to assess whether the large water body in one of the communities will result in differences in the intensity of malaria transmission between the two communities, and whether anti-sporozoite antibody seroprevalence and seroconversion rate estimates will be able to identify these differences. Estimates of seroconversion and seroreversion rates from the reversible catalytic model are based on a set of assumptions, one of which is that all individuals in a community, irrespective of age and frequency of exposure to infectious bites, sero-revert to being specific antibody-negative at the same rate [20]. In the current study, the superinfection model [21], which is a modification of the reversible catalytic model and accounts for the effect of repeated infections on the decay rate of antigen-specific antibodies, was used to assess any changes in transmission intensity in the two study communities (Gowrie/Vea and Soe). The ultimate aim of the study was therefore to determine whether sero-epidemiological models based on seroprevalence of antibodies against sporozoite antigens could assess short term differences in transmission between the two seasons with different disease transmission dynamics.

## Methods

### Ethics statement

Ethical and scientific approvals for the study were granted by the Institutional Review Boards (IRB) and the Scientific and Technical Committees (STC) of both the Noguchi Memorial Institute for Medical Research (NMIMR, Study number 108/11-12) and the Navrongo Health Research Centre (NHRC, study number NHRCIRB142). Both NMIMR (FWAA00001824) and NHRC (FWA00000250) hold a United States Government Federal Wide Assurance from the Office for Human Research Protections. Study participants or their parents/legal guardians gave written informed consent before enrollment into the study.

### Study sites and sample collection

**Fig 1:**
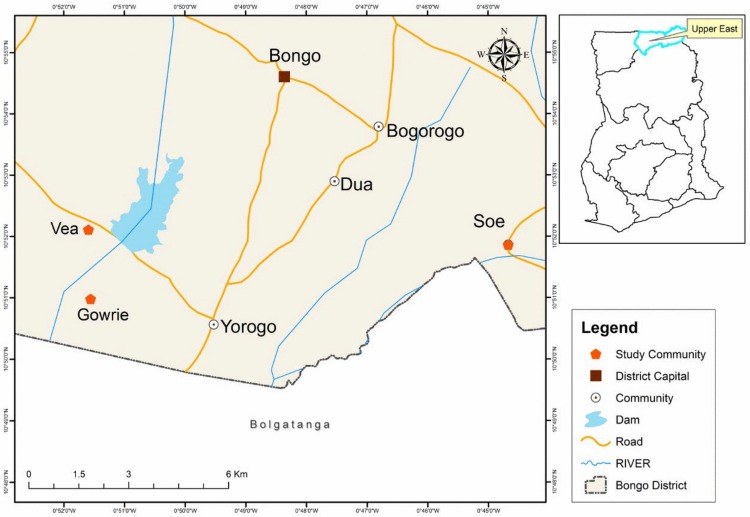
Map of the study area showing the dam and non-dam study communities. Credit: Mr. Richard Adade, GIS & Remote Sensing Unit, Department of Fisheries and Aquatic Sciences, Center for Coastal Management, University of Cape Coast, Cape Coast, Ghana.

The study was conducted as part of an on-going immunological study in the Gowrie/Vea and Soe communities in the Bongo district of the Upper East region of Ghana (Fig 1). The Bongo district, like a few other districts in the region, has been mapped by the demographic surveillance system of the Navrongo Health Research Centre and the database is regularly updated. The study areas have marked seasonal malaria transmission that overlaps with rainfall and vector distribution patterns. The peak rainfall season has traditionally been from May to November, followed by a period of very dry conditions from December to April the following year. Malaria transmission in the two study communities is therefore high from June through November and comparatively lower between January and April.

The Gowrie/Vea community has a large water body, the Vea irrigation dam, for agricultural purposes while the Soe community is about 20 km away from the Vea dam. The proximity of the water body to one of the two study communities is expected to result in differences in transmission of a mosquito-borne disease such as malaria. A questionnaire-based study that assessed the impact of the Vea dam on the health of individuals in communities surrounding the dam reported participant-perceived increases in the prevalence of a number of water-associated diseases such as malaria and schistosomiasis [22]. Aside the presence of the dam, the two study communities are largely rural with similar ecology and most inhabitants are subsistence farmers.

Individuals living in the two selected communities were recruited for the study. A cross-sectional community-based survey was conducted close to the end of the rainy season in November/December 2012 and a second similar survey was conducted at the end of the dry season in April 2013 in both study communities. Study participants were screened for sexual and asexual stage parasitaemia by light microscopic examination of thick blood smears at each cross-sectional time point. Venous blood (2 to 3 ml) was drawn from participants to prepare plasma samples for serology and these were stored at -20°C until use. Basic clinical and demographic data of participants were also captured by questionnaire to aid data analysis and interpretation. Samples from 609 participants between one and thirty years were analyzed in this study.

### Antigens and ELISA

Antigens used in this study were all from the 3D7 clone of *P*. *falciparum* and were expressed and purified under GMP conditions. The near full-length CSP protein containing 19 of the 38 NANP repeats was expressed in an *Escherichia coli* system [23]. The CelTOS protein is comprised of 174 amino acids including an N-terminal six-histidine tag within a 16-amino acid linker [24]. The AMA1 protein is comprised of amino acids 83 to 531 of the AMA1 ectodomain and was also expressed in *E*. *coli* [25,26].

Plasma levels of antibodies to the three parasite antigens were determined by a previously described indirect ELISA protocol [10]. Briefly, antigen-coated and blocked 96-well ELISA plates (Maxisorp, NUNC, Denmark) were incubated with 100 μl/well of duplicate test plasma samples diluted 1:1,000 in 1% non-fat milk in PBS. A pool of semi-immune sera was used as a standard calibrator (diluted 50 times for CSP, CelTOS and 5,000 times for AMA1 antigen plates) and titrated on each plate as an internal control. Plasma samples were incubated for two hours at room temperature and the plates subsequently developed with goat anti-human IgG conjugated to horseradish peroxidase (Invitrogen, CA, USA) followed by the substrate TMB (KEM-EN-TEC, Taastrup, Denmark). The colour reaction was stopped by the addition of 50 μl/well of 0.2 M H_2_SO_4_ and optical densities (ODs) were subsequently read at 450 nm using a 96-well ELISA plate reader (BioTek, VT, USA). In between all incubation steps, plates were washed five times with PBS, pH 7.4, 0.05% Tween 20 using an automated plate washer.

### Statistical and data analysis

For each measured antibody, OD data were normalized against the internal assay control. A mixture model was then fitted to the normalized and log_10_-transformed OD data from both communities over the two seasons to define a common cut-off value above which samples from both study communities were deemed antibody-positive as previously described [27]. Briefly, the distribution of log_10_-transformed OD values was fitted as the sum of two Gaussian distributions using maximum likelihood methods; one of the two distributions represents the seronegative population and the other represents the seropositive population. For each antigen, the mean log_10_-OD of the Gaussian corresponding to the seronegative population plus log_10_-three standard deviations was calculated, back-transformed and used as the cut-off for seropositivity. Seroprevalence was calculated as the proportion of samples with OD above this cut-off.

For each survey, the mean annual seroconversion rate (λ), which represents exposure to malaria over time and is analogous to the force of infection, and the mean annual seroreversion rate (ρ), the rate at which seropositive individuals revert back to seronegativity, were estimated using the superinfection model as described by Bosomprah *et al*. [21]. This model which is a modification of the reversible catalytic model, accounts for the fact that antibody responses can be boosted in individuals who are already seropositive. The mean annual λ and ρ were fitted using maximum likelihood methods that assume a binomial error distribution. Seroprevalence curves were plotted using observed seropositivity data and the median age of ten age groups, each of which is a decile of the observed data. The fitted values and 95% confidence limits were also plotted and the resulting λ is presented for each survey time point. Evidence for temporal changes in λ was explored by fitting models in which λ is allowed to change at a single time-point. Alkaike’s information criterion (AIC) was used to assess the significance of the change in λ against models with no change. Unlike the likelihood test, the AIC penalizes for the loss in degree of freedom. The model with the smallest AIC was considered to fit the data better. Profile likelihood plots were used to determine the most likely time that a change in λ, and hence transmission intensity, occurred.

For comparison of antibody levels between communities and sampling time points, OD data was converted into titres in arbitrary units (AU) using the 4-parameter logistic curve fitting program known as ADAMSEL (version b038, Ed Remarque, The Netherlands) and based on the positive plasma pool used as calibrator on each plate. Differences in antigen-specific antibody levels were assessed by comparing antibody levels (AU) in an age stratified manner (1–5 years, 6–15 years and 16–30 years). For each antigen, the Mann-Whitney U test was used to compare antibody levels between the two transmission seasons and between communities. The two-sample test for equality of proportions, with continuity correction, was used to compare proportions of parasite-positive individuals either between the two communities or between the two survey time points.

Analysis of data and graphics were performed using Stata (Statacorps, TX, USA) and R (version 3.2.2, R development Core Team). Differences were considered statistically significant when p values were less than 0.05.

## Results

### Study participant distribution and parasite prevalence

Plasma samples from a total of 609 study participants between one and thirty years were analyzed in this study. Of this number, 300 participants were from the rainy season survey (134 from Gowrie/Vea, the dam site, 166 from Soe, the non-dam site) and 309 from the dry season survey (147 from the dam site, 162 from the non-dam site). Study participants were categorized into three age groups, namely 1–5 years, 6–15 years and 16–30 years, and the proportions of individuals who had parasites, determined by light microscopy, were compared between the rainy and dry season for each age group. In the Gowrie/Vea community (dam site), parasite prevalence was significantly higher during the rainy season in the 1–5 year old (rainy season = 42%, dry season = 15.9%, p <0.0001, two-sample test for equality of proportions) and 16–30 year old (rainy season = 34.3%, dry season = 2.2%, p <0.0001) groups; there was no difference in parasite prevalence between the two seasons in the 6–15 year old group (rainy season = 30.6%, dry season = 31, p = 1). In the Soe community (non-dam site) however, parasite prevalence, in all three age groups, was at least two times higher in the rainy season compared to that of the dry season (Table 1). Proportions of parasite-positive individuals were however not significantly different between the two communities for all age groups per season (p > 0.05 in all cases), except for the higher proportion of parasite-positive 6–15 year old individuals in Soe (48.1%) compared to the proportion within the same age group in Gowrie/Vea (30.6%, p = 0.017). Further to this, for slide-positive individuals, there were no significant differences in median parasite density either between study communities or survey time points for any of the age groups (p > 0.05 in all cases).

**Table 1:** Comparison of parasite prevalence between surveys.

Community	Age group	% Parasite prevalence (n)		P value
		Rainy season	Dry season	
Gowrie/Vea (Dam site)	1–5 years	42 (50)	15.9 (44)	< 0.0001
	6–15 years	30.6 (49)	31 (58)	1
	16–30 years	34.3 (35)	2.2 (45)	< 0.0001
Soe (Non-dam site)	1–5 years	38 (58)	17.5 (57)	0.002
	6–15 years	48.1 (54)	22 (59)	0.0002
	16–30 years	30 (54)	4.3 (46)	< 0.0001

Sample size (n) for each age category is indicated in brackets.

### Antigen-specific antibody levels and seroprevalence amongst study participants

Levels of antibodies, expressed as the titre in arbitrary units, were also compared between the two survey time points in an age-stratified manner. Median anti-CSP antibody titres in the rainy season were about seven to 17-fold higher compared to corresponding titres in the dry season for all age groups in the two communities (p < 0.0001 in all cases, Table 2). Anti-CelTOS antibody titres were also about two to five-fold higher during the rainy season for all age groups in the two communities (p < 0.0001 in all cases). Anti-AMA1 antibody titres were only significantly higher in the rainy season for the 1–5 year old group in Gowrie/Vea (the dam site, p = 0.039). Thus generally, antibody levels against the sporozoite-specific antigens (CSP and CelTOS) were higher during the rainy season compared to their levels in the dry season (Table 2).

**Table 2:** Comparison of antigen-specific antibody levels between survey periods.

Community	Antigen	Age group (years)	Antibody levels (Arbitrary units)		P value
			Rainy season	Dry season	
Gowrie/Vea (Dam site)	AMA1	1–5	5312.8 (54.9–27712.8)	1569.9 (20.0–34877.9)	0.039
		6–15	10179.1 (255.6–28046.0)	9136.1 (145.7–49456.8)	0.93
		16–30	7157.0 (703.4–30361.1)	11899.0 (72.4–44888.4)	0.33
	CelTOS	1–5	346.0 (70.0–1203.1)	99.9 (19.7–864.6)	< 0.0001
		6–15	596.4 (66.9–4631.5)	191.2 (35.9–6237.7)	< 0.0001
		16–30	707.4 (151.2–3445.1)	178.8 (15.8–2469.7)	< 0.0001
	CSP	1–5	698.2 (120.4–9809.1)	41.4 (2.1–688.7)	< 0.0001
		6–15	1672.0 (161.8–17534.6	164.3 (14.9–1044.8)	< 0.0001
		16–30	2476.6 (210.7–36554.9)	236.3 (44.9–2272.6)	< 0.0001
Soe (Non-dam site)	AMA1	1–5	3391.7 (73.1–26082.1)	722.9 (17.9–32022.0)	0.51
		6–15	12979.0 (142.2–27353.0)	9831.8 (80.8–43404.4)	0.85
		16–30	9056.7 (107.2–29156.7)	11469.0 (100.0–42390.1)	0.54
	CelTOS	1–5	222.2 (8.0–2830.8)	108.6 (10.9–492.1)	< 0.0001
		6–15	648.7 (20.0–3100.0)	130.0 (20.5–1205.1)	< 0.0001
		16–30	577.3 (7.0–13688.2)	186.0 (9.1–2483.1)	< 0.0001
	CSP	1–5	503.1 (55.0–10424.1)	56.4 (2.1–444.6)	< 0.0001
		6–15	1271.6 (245.5–9336.1)	151.7 (10.7–1479.1)	< 0.0001
		16–30	2070.7 (155.5–13874.5)	300.0 (35.8–1827.6)	< 0.0001

Antibody levels expressed in arbitrary units and presented as median levels (min.–max.). P values obtained after Mann-Whitney test.

Comparison of antibody titres between the two communities showed that the median titre of anti-CSP antibodies in the younger age group (1–5 years) were statistically significantly higher in Gowrie/Vea (Dam site) compared to the median titre in the same age group from Soe (Non-dam site) (p = 0.024. Mann-Whitney U test, Table 2); the median antibody titre in the other age groups (6–15 years, 16–30 years) as well as median titres against the two other antigens (AMA1 and CelTOS) were however not different between the two communities.

**Fig 2:**
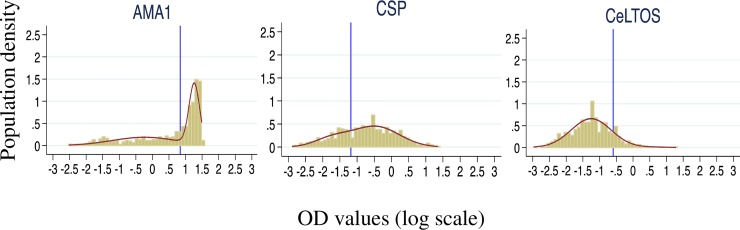
Distribution of seronegative and seropositive populations per antigen. For each antigen, the log_10_-transformed OD data from the two communities over the two seasons were combined and fitted to a mixture model. The mean log_10_-OD of the Gaussian corresponding to the seronegative population plus log_10_-three standard deviations was calculated, back-transformed and used as the cut-off for antigen-specific antibody seropositivity based on the distribution. Blue vertical lines in each case represent the estimated positivity cut-off values.

For each antigen-specific antibody, the log_10_-transformed OD data was fitted to a mixture model in order to define antigen-specific seropositive and seronegative populations (Fig 2). Anti-CSP antibody seroprevalence in both communities was significantly higher during the rainy season (Gowrie/Vea seroprevalence = 87.1%, 95%CI = 80.2–91.9%; Soe seroprevalence = 81.9%, 95%CI = 75.3–87.1%) compared to that of the dry season (Gowrie/Vea seroprevalence = 54.4%, 95%CI = 46.3–62.4; Soe seroprevalence = 49.1%, 95%CI = 41.4–56.8%). Seroprevalence of anti-CelTOS antibodies in both communities were also higher in the rainy season (Gowrie/Vea seroprevalence = 23.5%, 95%CI = 17.0–31.5; Soe seroprevalence = 28.3%, 95%CI = 21.9–35.7) compared to the dry season (Gowrie/Vea seroprevalence = 5.4%, 95%CI = 2.7–10.6%; Soe seroprevalence = 6.2%, 95%CI = 3.4–11.2, Table 3). Seroprevalence of anti-AMA1 antibodies were however not different between the two survey time points for any of the two communities (Table 3). Thus seroprevalence of antibodies against the two sporozoite-specific antigens were always higher in the rainy season while seroprevalence of antibodies to the classical blood stage antigen AMA1 did not differ between the rainy and dry seasons. For all three antigens, there were no differences in antibody seroprevalence between the two study communities at any of the two survey time points.

**Table 3:** Annual seroconversion and seroreversion rate estimates by the superinfection model based on antigen-specific antibodies.

Antigen	Survey	Site	Number of subjects (N = 609)	% seropositive (95%CI) *	Seroconversion rate (95%CI)	Reversion rate (95%CI)
AMA1	Rainy season	Dam	134	61.4 (52.7, 69.3)	0.354 (0.180, 0.696)	0.299 (0.128, 0.698)
		No Dam	166	64.5 (56.8, 71.4)	0.369 (0.213, 0.639)	0.273 (0.137, 0.541)
		Both	300	63.1 (57.4, 68.4)	0.360 (0.236, 0.550)	0.282 (0.165, 0.480)
	Dry season	Dam	147	53.1 (44.9, 61.0)	0.189 (0.098, 0.365)	0.179 (0.071, 0.451)
		No Dam	162	56.5 (48.7, 64.0)	0.224 (0.127, 0.396)	0.194 (0.088, 0.427)
		Both	309	54.9 (49.3, 60.4)	0.208 (0.135, 0.321)	0.189 (0.104, 0.344)
CeITOS	Rainy season	Dam	134	23.5 (17.0, 31.5)	0.040 (0.019, 0.086)	0.064 (0.011, 0.374)
		No Dam	166	28.3 (21.9, 35.7)	0.071 (0.035, 0.144)	0.135 (0.047, 0.393)
		Both	300	26.2 (21.5, 31.5)	0.055 (0.032, 0.092)	0.099 (0.040, 0.248)
	Dry season	Dam	147	5.4 (2.7, 10.6)	0.008 (0.001, 0.042)	0.064 (0.001, 3.281)
		No Dam	162	6.2 (3.4, 11.2)	0.007 (0.002, 0.031)	0.033 (0.000, 7.559)
		Both	309	5.8 (3.7, 9.1)	0.007 (0.002, 0.022)	0.045 (0.002, 1.217)
CSP	Rainy season	Dam	134	87.1 (80.2, 91.9)	0.500 (0.292, 0.857)	0.121 (0.036, 0.410)
		No Dam	166	81.9 (75.3, 87.1)	0.359 (0.237, 0.545)	0.077 (0.025, 0.240)
		Both	300	84.2 (79.6, 88.0)	0.414 (0.300, 0.571)	0.094 (0.042, 0.211)
	Dry season	Dam	147	54.4 (46.3, 62.4)	0.085 (0.052, 0.138)	0.002 (0.000, 3.270)
		No Dam	162	49.1 (41.4, 56.8)	0.072 (0.044, 0.118)	0.001 (0.000, 2.630)
		Both	309	51.6 (46.0, 57.2)	0.078 (0.055, 0.110)	0.001 (0.000, 6.680)

*For each antigen, a seropositivity cut-off was estimated using combined log_10_-transformed antibody OD data from both communities over the two survey time points (calculated as the back-transformed log_10_-mean OD of the negative distribution + log_10_-three times the standard deviation, Fig 2).

### Antigen-specific seroconversion and seroreversion rates

**Fig 3:**
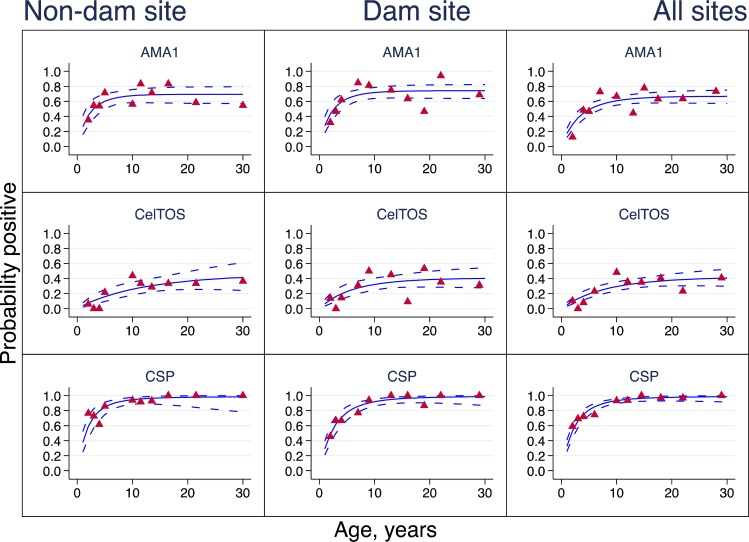
Seroprevalence curves for rainy season survey. Seroprevalence curves represent the rate at which individuals become seropositive to specific antigens. In each graph, points represent age seroprevalence (divided into deciles), unbroken lines represent maximum likelihood curves and broken lines represent 95% confidence intervals.

**Fig 4:**
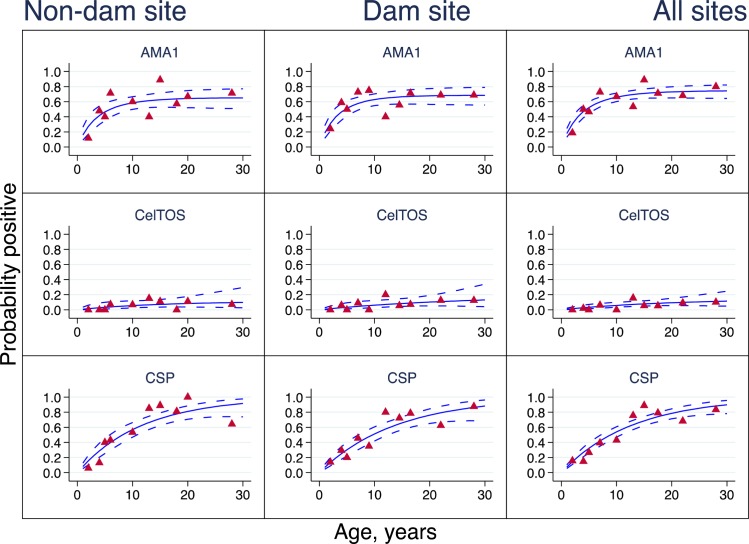
Seroprevalence curves for dry season survey. Seroprevalence curves represent the rate at which individuals become seropositive to specific antigens. In each graph, points represent age seroprevalence (divided into deciles), unbroken lines represent maximum likelihood curves and broken lines represent 95% confidence intervals.

Antigen-specific seroprevalence curves for the rainy and dry seasons are presented in Figs 3 and 4 respectively, and seroconversion and seroreversion rate estimates are provided in Table 3. All seroprevalence curves were monophasic, suggesting that no changes in λ were observed within the two communities based on antibody seroprevalence data collected either during the rainy or dry season. A comparison of λ between survey time points however showed a statistically significant difference in λ between the rainy and dry season estimates for anti-CSP antibodies in both study communities and for anti-CelTOS antibodies in the non-Dam community (Soe) alone (Figs 3 and 4, Table 3). Anti-AMA1 antibody λ was not different between survey time points in both communities. For all antigen-specific antibodies, there were no significant differences in λ between the two study communities at any of the two survey time points (Table 3).

A comparison of antigen-specific λs based on the combined data showed that rainy season anti-AMA1 λ (0.360, 95%CI 0.236–0.550) was statistically similar to that of anti-CSP λ (0.414, 95%CI 0.300–0.571), and both were significantly higher than that of anti-CelTOS antibodies (λ = 0.055, 95%CI 0.032–0.092). During the dry season, anti-AMA1 antibody λ for the combined data (0.208, 95%CI 0.135–0.321) was significantly higher than anti-CSP λ (0.078, 95%CI 0.055–0.110) and anti-CelTOS λ (0.007, 95%CI 0.002–0.022). Anti-CSP λ was also significantly higher than anti-CelTOS λ during both the rainy and dry seasons (Table 3). These collectively suggest that antibodies against AMA1 and CSP form at a significantly faster rate than those against CelTOS during the rainy season, but anti-CSP antibody formation rate also reduces significantly compared to anti-AMA1 antibodies during the dry season.

Estimates of ρ for all three antigen-specific antibodies measured were statistically similar to the corresponding λ estimates for the same antigens, with the exception of the combined rainy season data for anti-CSP antibodies (λ = 0.414, 95%CI 0.300–0.571; ρ = 0.094 (0.042–0.211, Table 3). While this difference in anti-CSP λ and ρ estimates point to a potential difference in the rates of formation and decay of anti-CSP antibodies, this data comparison generally suggests that antibody levels are maintained over time since they are formed and degraded at similar rates. There were also no significant differences amongst the antigen-specific antibody ρ across communities and survey seasons (Table 3).

Currently available models are designed to estimate the annual rates of seroconversion and seroreversion, and the single λ and ρ values that are estimated as outputs may not reflect the earlier observed changes in antibody levels and seroprevalence between the two survey time points within the same year. Thus with the current design of these models, seroconversion and seroreversion rate estimates may not detect the observed changes in antigen-specific antibody levels and seroprevalence between the two sampling periods.

## Discussion

Malaria elimination is high on the agenda of control programmes in many malaria-endemic countries and a continued sustenance of control efforts as well as effective monitoring of disease transmission is necessary in these areas to ensure success. There are a number of proven malaria controls tools which continue to be deployed in areas where transmission is believed to still be on-going, but these currently available tools for monitoring transmission and the success of such control efforts have serious limitations, especially when transmission levels fall below the detection limits of these tools [3,20,28]. There is therefore the need to develop more sensitive tools for monitoring low levels of transmission to ensure successful disease elimination and prevent the resurgence of severe disease in previously semi-immune individuals due to declining immunity as a direct result of declining transmission [29–31]. Transmission monitoring is also important as it can inform about the appropriate timing of control interventions such as drug and vaccine trials. Mathematical models that estimate the rate of antibody seroconversion against mostly blood stage parasite antigens are gaining relevance [2,14,27,32], but a difficulty with this approach has been the identification of appropriate antigen targets since the levels of specific antibodies to these targets must reflect any changes in transmission patterns [20].

Using the reversible catalytic model, we have previously demonstrated the usefulness of the seroprevalence of anti-CSP antibody as a marker for malaria transmission intensity in an area of very low transmission in Ghana [10]. Using this model, the short-lived nature of anti-CSP antibodies was demonstrated by the observation that these antibodies developed at a slower rate compared to antibodies to the blood stage antigen AMA1 but waned at a significantly faster rate than anti-AMA1 antibodies [10]. In the current study, we sought to further assess these models for estimation of short term changes in transmission by measuring and comparing specific antibody levels against CSP, CelTOS and AMA1 in the plasma of individuals living in an area of seasonal malaria transmission and estimating the seroconversion and seroreversion rates of these specific antibodies.

Parasite prevalence was generally not different between the two communities but was significantly higher in all age groups during the rainy season when compared to the dry season in both communities (Table 1). The only exception to this was the similar parasite prevalence levels in the 6–15 year old group between the rainy and dry seasons at the dam site (Table 1). While this generally reflects the higher preponderance of vectors and hence the extent of disease transmission during the rainy season, the observed high parasite prevalence in the 6–15 year old group suggests a greater exposure of this age group to parasites. Similar findings of high parasite prevalence in this age group have been observed and reported elsewhere [33–36].

Antibody levels against the two sporozoite-specific antigens (CSP, CelTOS) were higher during the rainy season when compared to their levels during the dry season for all age groups in the two communities. These changes reflected the pattern of transmission and support the proposed use of anti-sporozoite antibodies as transmission monitoring markers [10]. With the exception of higher rainy season anti-AMA1 tires in the 1–5 year old group at the dam site, anti-AMA1 antibody titres were similar between seasons for all other age groups within the two communities. Thus anti-AMA1 antibody levels did not reflect the change in transmission over the two clearly marked seasons, making these antibodies less suitable for monitoring short term changes in transmission intensity. Antibody levels against all three antigens were generally comparable between the two communities for all age groups.

Despite the lack of differences in antibody seroprevalence between the two communities for any of the age groups compared, the antibody seroprevalence data presented confirms the potential utility of anti-sporozoite antibodies as surrogate markers for assessing short term changes in malaria transmission intensity. Anti-sporozoite antibody levels were high during the rainy season and decreased significantly to low levels in the dry season (Table 3). Anti-AMA1 antibody seroprevalence was not different between seasons, confirming the persistence of these antibodies and the reduced likelihood of anti-AMA1 antibodies fluctuating with short term changes in transmission intensity [20,37].

The superinfection model, which corrects for the effect of repeated exposures on the rate of antibody decay [21], was used to fit seroprevalence data for the estimation of seroconversion and seroreversion rates. While no changes in λ were observed for cross-sectional data from any of the communities or sampling time points (Figs 3 and 4), it was generally observed that anti-AMA1 and anti-CSP antibodies seroconverted at statistically similar rates during the rainy season but anti-CSP antibody seroconversion was significantly reduced during the dry season (Table 3). This directly supports the finding that the levels of anti-CSP antibodies, but not anti-AMA1 antibodies, are a good predictor of parasite exposure over time [11–13]. Indeed, anti-AMA1 antibodies seroconverted at similar rates during the rainy and dry seasons, while anti-CSP antibodies seroconverted at a significantly higher rate during the rainy season compared to the dry season (Table 3). These therefore collectively confirm the observations made by comparison of parasite prevalence and antibody levels between the two sampling time points. For all measured antibodies, the generally similar estimates for the rates of seroconversion and seroreversion (Table 3) suggest that antibody levels will most likely remain fairly consistent throughout the year.

The finding that no single cross sectional antibody seroprevalence data, including those of anti-CSP antibodies, was able to predict changes in malaria transmission intensity suggest that the estimation models based on dichotomized antibody data may not be optimal for assessing within year changes in malaria transmission. These antibody seroprevalence models are designed to predict annual changes in seroconversion rates by averaging the patterns of seropositivity changes over an entire year [20,21,32]. This may therefore preclude the possibility of detecting changes in transmission that occur within a 12-month period for areas with seasonal disease transmission. It would thus be important to develop models that can predict seasonal changes in malaria transmission, which would usually occur within four to six months. These dichotomized data-based models may therefore need modification to allow for short term, possibly monthly, estimation of seroconversion and seroreversion rates based on cross-sectional data. Fitting of antibody data to other models may also help improve on the predictive power of data from cross-sectional surveys. For example, recent sero-epidemiology studies have reported more precise parasite exposure rates based on new models that utilize continuous antibody data compared to seroconversion estimates by the catalytic model based on dichotomized antibody data [32,38].

There is also the need to look for additional sporozoite antigens with similar or better transmission monitoring properties, especially since the CSP-based malaria vaccine RTS,S stands the chance of being approved for human use. For communities that may potentially this vaccine, the levels of blood anti-CSP antibodies may be contributed by the vaccine as well as by natural infection and may therefore not necessarily reflect the extent of malaria transmission. A recent study screened over 850 *Plasmodium* antigens using microarrays and identified a limited number of proteins, including parasite-specific enzymes, that were strongly associated with either short term exposure to parasites or the incidence of clinical disease in individuals in an endemic setting [5].

In summary, the levels and seroprevalence of antibodies against sporozoite antigens may be relevant surrogate markers for monitoring changes in malaria transmission patterns in areas of seasonal disease transmission. The levels and seroprevalence of sporozoite-specific antibodies, especially those against the CSP antigen directly reflected changes in malaria transmission between the rainy and dry seasons. Seroconversion rate estimates for all three assessed markers however did not reflect the marked changes in transmission intensity within a year, and this is most likely due to the fact that the prediction model used is designed to estimate changes occurring over an entire year. It is possible that analysis of data with other models that account for some of the basic assumptions upon which the reversible catalytic and superinfection models are based may yield more detailed and robust data to support this approach to malaria transmission estimation.
